# Phase Transformation Kinetics During Post-Weld Heat Treatment in Weldments of C-250 Maraging Steel

**DOI:** 10.3390/ma18122820

**Published:** 2025-06-16

**Authors:** Mercedes Andrea Duran, Pablo Peitsch, Hernán Gabriel Svoboda

**Affiliations:** 1 Facultad de Ingeniería, Universidad de Buenos Aires, GTSyCM3, Buenos Aires C1053ABH, Argentina; hsvobod@fi.uba.ar; 2 Laboratorio de Investigación Aplicada a la Producción y el Trabajo, Universidad Nacional de Hurlingham (UNAHUR), Hurlingham B1688AXC, Argentina; 3 División de Transformaciones de Fase, Gerencia Materiales, Comisión Nacional de Energía Atómica, Buenos Aires C1429BNP, Argentina; pablo.r.peitsch@gmail.com; 4 Instituto de Tecnologías y Ciencias de la Ingeniería (INTECIN), CONICET-Universidad de Buenos Aires, Buenos Aires C1120AAQ, Argentina

**Keywords:** maraging steels, welding, post-weld heat treatment, precipitation, reverted austenite

## Abstract

Welding of maraging steels leads to a microstructural gradient from base material (BM) to weld metal (WM). During post-weld heat treatment (PWHT) the precipitation and reverted austenite (γ_r_) reactions will occur defining the mechanical properties. These reactions are affected by the microstructure and local chemical composition of each zone in the “as welded” (AW) condition. This effect has not been clearly described yet nor the evolution of the microstructure. The objective of this work was to analyse the phase transformations at the different zones of the welded joint during the PWHT to explain the microstructure obtained at each zone. Samples of C250 maraging steel were butt-welded by GTAW-P (Gas Tungsten Arc Welding—Pulsed) process without filler material. The AW condition showed an inhomogeneous microhardness profile, associated with a partial precipitation hardening in the subcritical heat affected zone (SC-HAZ) followed by a softening in the intercritical (IC-HAZ) and recrystallized heat affected zone (R-HAZ). A loop-shaped phase was observed between low temperature IC-HAZ and SC-HAZ, associated with γ_r_, as well as microsegregation at the weld metal (WM). The microstructural evolution during PWHT (480 °C) was evaluated on samples treated to different times (1–360 min). Microhardness profile along the welded joint was mostly homogeneous after 5 min of PWHT due to precipitation reaction. The microhardness in the WM was lower than in the rest of the joint due to the depletion of Ni, Ti and Mo in the martensite matrix related with the γ_r_ formation. The isothermal kinetics of precipitation reaction at 480 °C was studied using Differential Scanning Calorimetry (DSC), obtaining a JMAK expression. The average microhardness for each weld zone was proposed for monitoring the precipitation during PWHT, showing a different behaviour for the WM. γ_r_ in the WM was also quantified and modelled, while in the IC-HAZ tends to increase with PWHT time, affecting the microhardness.

## 1. Introduction

Maraging (MA) steel is an ultra-high strength alloy of the family 18% Ni, with extra-low carbon content (<0.003), a low but important content of Ti [1]. The C250 grade also contains Co. It is widely used in the manufacturing of Solid Rocket Motor (SRM) cases as a result of a good combination of ultra-high tensile strength (in the order of 1700 MPa), excellent fracture toughness and weldability [2,3,4]. The main strengthening mechanism is precipitation, which is produced during a low temperature ageing heat treatment (440–520 °C, 3–5 h). The high density of dislocation present in the martensite matrix allows a fine distribution of nano-size precipitates (<30 nm) such as Ni_3_(Mo, Ti) and Fe_2_Mo [5,6]

SRM cases are mainly fabricated by welding [4]. The MA-C250 steel is commonly used in annealed condition, followed by post-weld heat treatment (PWHT) to adjust the final mechanical properties [7,8]. As a consequence of weld thermal cycles established in the “As welded” (AW) condition a microstructural gradient is produced from the weld metal (WM) to the base material (BM). The phase transformations during the welding process can occur by a number of simultaneous mechanisms such as shear, short-range diffusion or diffusion controlled nucleation and growth [9]. Therefore, the peak temperatures and cooling rates attained in each zone will define the final microstructure along the whole welded joint. In the WM the obtained microstructure is a soft martensite, with microsegregated zones, rich in Mo, Ni and Ti [7,10] produced during the solidification process, following celular-dendritic pattern. In the HAZ (Heat Affected Zone), in the regions where temperatures above A_c3_ were reached, a fully austenitic structure was formed in the heating, which transformed into a full fresh martensite matrix during cooling to room temperature. Near the fusion line, grain growth takes place (CG-HAZ), as a consequence of the high peak temperatures achieved, while towards colder temperatures (near A_c3_) the grain is refined (FG-HAZ). Some authors have called this region the light zone [7,11,12]. When the MA-C250 steel reaches peak temperatures between A_c3_ and A_c1_ (Intercritical HAZ: IC-HAZ), partial austenitization takes place during heating and the microstructure at room temperature is composed of fresh and partially aged martensite. Furthermore, some authors have pointed out a region named Dark Zone (DZ), associated with the material that was exposed between 590–740 °C (around A_c1_), promoting the reversion of martensite into depleted martensite and reverted austenite (γ_r_), which will not transform to martensite on cooling to room temperature [7,8,10,11,12,13,14]. The fraction formed depends mainly on the welding heat input [7,8,11,15,16]. Finally, there is the Subcritical HAZ (SC-HAZ) which has been reheated below A_c1_. Due to the high Co content, the time for the precipitation reaction is reduced, causing a slight precipitation in the IC/SC-HAZ after welding of MA steel [7,11].

The ageing of the martensite throughout the welded joint is the main reaction that takes place during PWHT. The strength and microhardness of the weld joint increases due to the precipitation of intermetallic compounds, mainly Ni_3_(Mo, Ti) [17,18]. Several articles have studied the precipitation and austenite reversion reactions in MA steel [1,5,6,19,20,21] and its effect on the mechanical properties of the BM [1,5,6,7]. Due to the very small size of the precipitates, its observation is quite difficult and must be used non-conventional techniques such as high resolution Transmission Electron Microscopy (TEM) [1,5,22]. It has been reported that dislocations in the martensite matrix are preferential nucleation sites for precipitates, generating a uniform distribution of them throughout the matrix [5,6,9,23]. Some authors have used Differential Scanning Calorimetry (DSC) technique to study the precipitation and martensite reversion reaction in a MA-C250 maraging steel obtaining the JMAK parameters for isothermal ageing kinetics for different temperatures [1,19,24]. These studies have been mainly applied to BM, where the effect of the grain size, dislocation density, microsegregation, partial precipitation or γ_r_ presence, associated with AW condition microstructure, on the precipitation kinetics during PWHT has not been investigated.

The martensite reversion reaction is the other transformation that takes place during the PWHT of welded joints. The γ_r_ formation at the WM during PWHT is a phenomenon related to the microsegregation of Ni, Mo and Ti, produced during solidification, which results in a slightly lower hardness of this zone, compared with the rest of the joint [10,12,13]. Some authors have mentioned that in welding processes with filler material, the amount of γ_r_ could be higher than 10%, but it can be reduced and even eliminated by modifying the composition of the filler metal [12,15]. However, in autogenous welding (GTAW), the composition of the WM remains almost the same as the BM and can not be modified. The γ_r_ formed in the DZ as a consequence of the weld thermal cycle would also increase during PWHT [1,7,20,25,26] and could affect the final local hardness of this zone [17,27]. The study of the γ_r_ in welded joints and its evolution during PWHT in autogenous welds still needs a better description.

Therefore, the different final microstructure developed at each zone of a welded joint after PWHT would affect the mechanical properties of the welded component [7,14,15,16]. The discussion about the phase transformations that take place in the different zones of welded joints of MA steels is still not clear enough. In addition, there is a lack of information available about the microstructure evolution of weldments during PWHT.

The aim of the present work is to improve the comprehension of the phase transformations that take place in the different zones of MA-C250 steel welded joints, during PWHT at 480 °C. Also, the extension of methodologies and techniques used for base metals to the study of welded joints is an objective of this work.

## 2. Materials and Methods

### 2.1. Base Material

MA-C250 steel sheet with 2 mm thickness in annealed condition (AR: as received) was studied in this work. The measured chemical composition of the material, determined by Optical Emission Spectrometry (OES) following the requirements of the standar ASTM E 415 [28], is shown in Table 1.

To characterise the BM, a sample in AR condition was prepared for metallographic observation. Additionally, as a reference, another sample was subjected to ageing treatment at 480 °C per 3 h respectively (HT).

For metallographic observation the samples were ground and polished. Modified Fry’s reagent (50 mL HCI, 25 mL HNO_3_, 1 g CuCl_2_ and 150 mL water) was used to etch the BM for metallographic analysis by Light Microscopy (LM).

**Figure 1:**
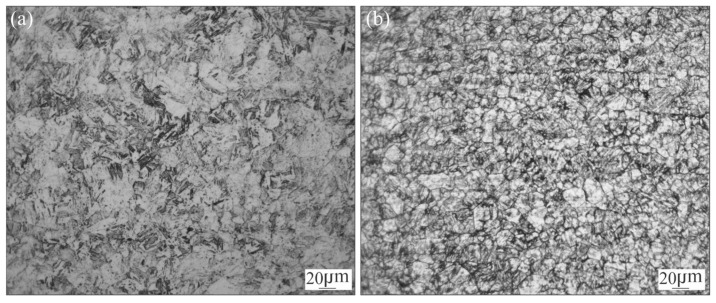
Optical microscopy of BM: AR (**a**) and HT (**b**) for MA-C250 steel.

The microstructure in annealed and aged conditions are shown in Figure 1. In annealed condition (Figure 1a), the material exhibits a homogenous fine grain size structure composed of soft martensite. After ageing treatment the material exhibits a structure composed of aged martensite (Figure 1b).

Vickers microhardness measurements (HV1) were performed for both AR and HT conditions. For AR the microhardness was 349 HV. After HT the hardness increased to 544 HV which is in accordance with the expected value for this material [1,15].

### 2.2. Welding

Coupons of 100 mm × 50 mm of the MA-C250 steel sheet were butt-welded by GTAW-Pulsed, without filler material and a single pass configuration. Ar was used as shielding (15 L/min) and Ar−20%CO_2_ as backing gas (15 L/min). Table 2 shows the welding parameters used. During the thermal welding cycle, voltage and current signals were acquired with an acquisition rate of 60,000 samples/s. The Root Mean Square Voltage Voltage (V_RMS_) and Root Mean Square Current (I_RMS_) values were obtained, and then, the heat input (HI) could be calculated by: HI = V_RMS_ ∗ I_RMS_/Welding Speed [29].

After welding, cross section samples extracted from the welded joint were subjected to a PWHT (P) at 480 °C, with different times of permanence: 1, 3, 5, 10, 15, 30, 60, 180 and 360 min.

### 2.3. Microstructural Characterization, Local Chemical Analysis and Microhardness Profiles

Cross section specimens of AW and different PWHT conditions were prepared for metallographic analysis. The samples were grounded, polished and etched following the same procedure described for the BM. The Light Microscopy (LM) and Scanning Electron Microscopy in secondary electron mode (SE-SEM) were used for macro and microstructural characterization. For AW conditions, local chemical composition at the WM was evaluated using Energy Dispersive X-ray Spectroscopy (EDS). In the samples subjected to PWHTs, the γ_r_ fraction in the WM was measured by quantitative metallography using ImageJ software (2024 version), considering ten images for each condition. Vickers microhardness (HV1) profiles were measured along the entire welded joint for all the analysed conditions, on the mid-thickness line, with a distance between measurements of 310 μm.

### 2.4. Differential Scanning Calorimetry (DSC)

Discs of 4 mm in diameter and 2 mm in thickness were extracted from BM using Electrical Discharge Machining (EDM) to study the phase transformations by DSC. Runs at different heating rates (HR) (20, 40, 60 and 80 °C/min) were done in a Setaram Labsys Evo calorimeter (Caluire, France) from room temperature up to a maximum of 850 °C. All the tests were performed under the Argon atmosphere, with a flow of 14 mL/min. The blank subtraction was carried out in all HR. The obtained thermograms were analysed and the observed phase transformations were discussed. Endothermic and exothermic reactions were identified based on previous works for this material [19]. For each reaction, the peak and temperature were defined.

As it was mentioned, Guo et al. [19] proposed a methodology to model the isothermal kinetics of the precipitation reaction in MA steels from the DSC results, obtained from continuous heatings, at different heating rates. In this work, it was used to study the precipitation reaction during PWHT (480 °C) of MA-C250.

Then, the exothermic peak of each DSC thermogram associated with the hardening precipitation reaction was analysed to obtain the transformed fraction f(T) as a function of temperature (*T*), according to Equation (1),

(1) $$f(T)=\frac{\int_{T_{0}}^{T}HdT}{\int_{T_{0}}^{T_{e}}HdT}$$

where *H* is the heat flow measured, $T_{0}$ and $T_{e}$ are the transformation onset and end temperatures [19,24]. The obtained at the start temperature was associated with 0 transformed fraction and the one for the finish temperature was fraction 1. The intermediate obtained values were normalized with this criterion, obtaining the transformed fraction as a function of temperature.

In order to calculate the kinetic model parameters, the transformation activation energy (*E_a_*) must be determined, according to the modified Kissinger method (Equation (2)) [19,24].

(2) $$ln\frac{T_{f}^{2}}{HR}=\frac{E_{a}}{RTf}+ln\frac{E_{a}}{RK_{0}}+ln\beta_{f}$$

where *T_f_* is the characteristic temperature for a given process, *HR* is the heating rate, *E_a_* the activation energy, *R* is the universal gas constant and *K*_0_ and *β_f_* are constants. Then, the *E_a_* [kJ/mol] could be obtained by a linear regression between $ln\;\frac{{T_{p}}^{2}}{HR}\;$ and $\frac{1000}{T_{p}}$ data), where *T_p_* is the temperature of the precipitation peak and *HR* is the heating rate used in each experiment. The slope of the line is $\frac{E_{a}}{R}$, from which *E_a_* can be obtained.

With this information, the precipitate transformed fraction was obtained by fitting the DSC using the modified JMAK (M-JMAK) equation for non-isothermal phase transformation kinetics [19,24] described in Equation (3).

(3) $$f=1-exp[-(\frac{k_{0}}{HR}\left(exp\frac{-E_{a}}{RT}\right)\left(T-T_{0}\right){)}^{n}]$$

where *n* is the Avrami exponent, *k*_0_ is the reaction rate and *T*_0_ is the onset temperature of the phase transformation determined from DSC curves. *k*_0_ and *n* coefficients were determined for each HR.

The average values of *k*_0_ and *n* values, also with *E_a_*, were used to determine the parameters for the precipitation during an isothermal ageing process using the JMAK equation (Equation (4)).

(4) $$f=1-exp[-{\left(kt\right)}^{n}]$$

where the constant *k* was calculated with the average *k*_0_ and *E_a_* using Equation (5) [19]. In this case it was analysed for an ageing temperature of 480 °C.

(5) $$k\left(T\right)=k_{0}exp(-\frac{E_{a}}{RT})$$

Following this methodology, the kinetics of isothermal precipitation for the BM was obtained from the DSC thermograms performed in continuous heating, at different heating rates. These results were evaluated at 480 °C and compared with the experimental data obtained from microhardness measurements during PWHT at this temperature.

## 3. Results and Discussion

### 3.1. Microstructural Characterization of Welded Joint

**Figure 2:**
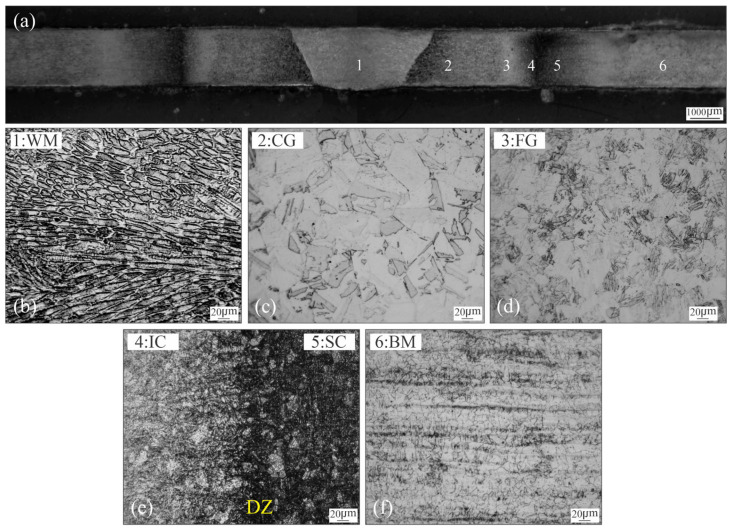
Cross section macrostructure of welded joint in AW condition (**a**) and corresponding microstructures: 1-WM (**b**), 2-CG-HAZ (**c**), 3-FG-HAZ (**d**), 4-IC-HAZ/5-SC-HAZ (**e**) and BM (**f**).

The cross-section micrograph of the weldment in AW condition is shown in Figure 2a, showing a full penetration. No macroscopic defects were observed.

Figure 2b–f show the microstructural variations generated after welding thermal cycle, from the weld centreline (WCL) to the BM. The WM represents the volume of material that has exceeded the melting temperature (T_m_). It shows columnar grains of martensite, with a cellular-dendritic solidification pattern (Figure 2b).

**Figure 3:**
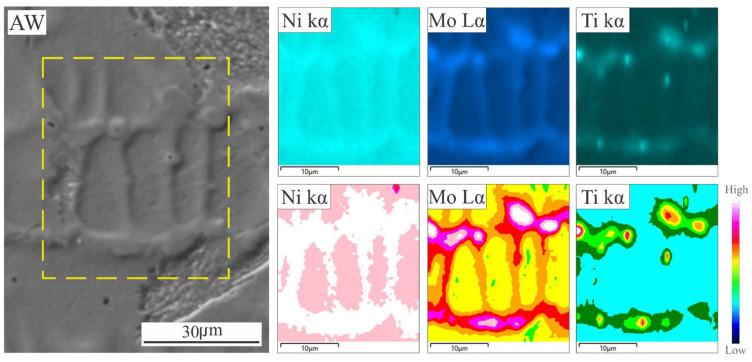
SE-SEM micrograph and elemental EDS and ”heat” mapping in the WM of the AW sample.

As a consequence of this unstable solidification front, microsegregation is expected to occur [7,9,14,15,16]. Figure 3 shows the elemental and “heat” mapping obtained by EDS and subsequent image processing for the WM in the AW condition. The heat map was obtained using Zeiss EDS software image processor (2023 version). Color variation is associated with the concentration of the considered element, providing an additional tool for the interpretation for the element distribution in the region being analyzed

It can be seen that there is a segregation of Ni, Mo and Ti to the cell boundaries as was previously found [10], in accordance with their partition coefficients [30].

**Figure 4:**
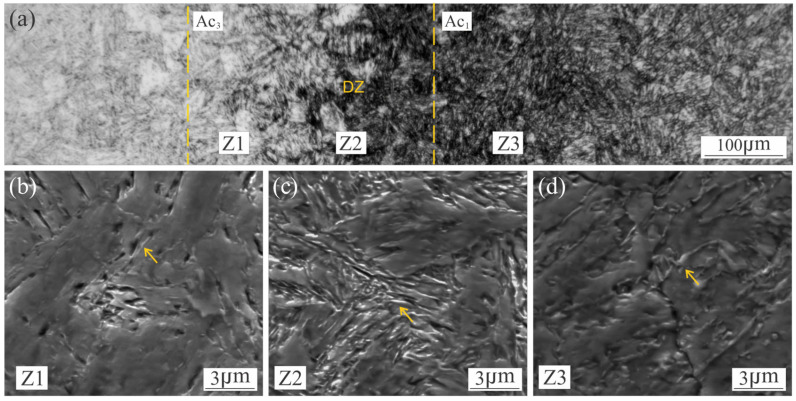
Microstructural evolution in HAZ of AW sample (**a**), SE-SEM detail of: Z1 (**b**), Z2 (**c**) and Z3 (**d**).

In the HAZ, different regions have been observed, corresponding with the peak temperature achieved [7,11]: CG-HAZ, FG-HAZ, IC-HAZ and SC-HAZ. The coarse grain fresh martensite microstructure takes place in the CG-HAZ (Figure 2c) due to the high temperature attained. Where the temperature reached was close above A_c3_ (approx. 750 °C) the FG-HAZ microstructure was formed by a grain refined fresh martensite. Figure 2e shows the IC-HAZ, where the material was exposed to temperatures between A_c1_ and A_c3_ (590–750 °C) [1,31], inducing the partial decomposition of the martensite into austenite and some partial ageing of the remaining martensite. A confined DZ (of 300 μm width approx.), could be observed which corresponds to the material reheated at temperatures close to A_c1_. Some authors have pointed out that DZ is the result of the high tendency of martensite reversion into austenite, due to the microsegregation produced in the martensite, generating Ni-Rich zones and promoting a fine dispersion of γ_r_ [14,18,20,23,31] and some precipitation at the remaining martensite [18]. Finally, previously to reach the BM (Figure 2f), it can be seen the SC-HAZ (Figure 2e), which has been heated to a temperature below A_c1_ but over the precipitation start temperature (approx. 440 °C), producing a partial precipitation [11]. Figure 4a shows a local micrograph corresponding to the darkened area, where the DZ can be observed, previously to the FG-HAZ.

As shown in Figure 4c an oriented loop-shaped phase was observed (yellow arrows), which can be identified as γ_r_ [20,23]. It can be seen that the presence of this phase extends beyond the DZ (Figure 4b,d), which could indicate that the microsegregation of martensite would start at temperatures below A_c1_, reaching the maximum of the martensite reversion in the DZ. Dos Santos et al. [23] have been pointed out that, when the MA steel is exposed at temperatures above to ~550 °C and close to A_c1_ or for prolonged times, a martensite reversion can be promoted as a consequence of the local enrichment of gamma-stabilising elements, such Ni, in the lath-lath interphase and grain boundaries. In accordance with other authors, they have mentioned that dislocations can contribute to the martensite reversion to γ_r_, increasing the substitutional-element mobility through pipe diffusion mechanism [9,20]. Li et al. [10] have mentioned that the phase is fine and regular with a size of 100 nm of width after 560 °C, 3 h.

**Figure 5:**
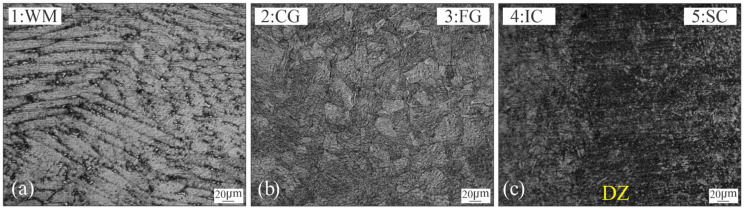
Microstructures of different weld zones for 3h-PWHT condition: WM (**a**), CG-HAZ (**b**), and IC/SC-HAZ (**c**).

Figure 5 shows the microstructures obtained after 3 h of PWHT in different zones of the welded joint.

In all cases, it can be seen a darkening of the microstructure associated with the precipitation. As a consequence of nanosize dimension (<30 nm), the precipitates can not be determined with LM nor SEM, being necessary the use of advanced microanalysis techniques to identify them [5,10]. Precipitation of an intermetallic phase like Ni_3_(Mo, Ti) type has been reported for MA-C250 after ageing at 480 °C-3 h [1,5,6]. The high content of Co limits the dissolution of Mo in the matrix and associated with the high partition coefficient of Ni, causes a martensite rich in precipitate-forming elements [6,9,32,33,34]. Nevertheless, for welded joints, the effect of PWHT could not be uniform. As was shown previously, in the AW condition, microsegregation, partial precipitation, γ_r_, recrystallization and grain growth (Figure 2) would affect the result of the PWHT.

**Figure 6:**
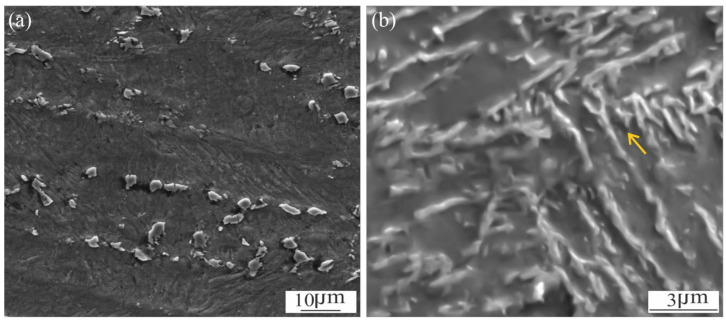
SE-SEM images of reverted austenite: WM (**a**) and DZ (**b**) in 3 h-PWHT condition.

Figure 5a shows the WM which is composed of islands of γ_r_ (white phase), dark etched regions around the γ_r_ and the aged martensite matrix structure. Figure 6a showed a detail of the γ_r_ islands dispersed across the WM.

It can be seen that the γ_r_ formation takes place at the intercellular-dendritic zone, as a consequence of the segregation of Ni, Mo and Ti in the last liquid to solidify (Figure 3), according to what was previously observed [8,10,18].

At the DZ, the loop-shaped phase is still observed after 3 h of PWHT, showing a slight thickening (Figure 6b), which was also previously found [7,11,12].

Tariq et al. [18] have shown that low fractions of γ_r_ tend to enhance in ductility without considerably reducing strength. In a previous work of the authors [13] a slight decrease of the local hardness at the DZ in the PWHT condition was measured, which could be associated with the γ_r_. A lower microhardness was also measured at WM microstructure, due to the presence of γ_r_.

The evolution of precipitation and reverted austenite formation with the duration of PWHT is analysed and discussed in the next sections, as well as its effects on microhardness along the whole welded joint.

### 3.2. Microhardness Evolution of the Welded Joint During PWHT

**Figure 7:**
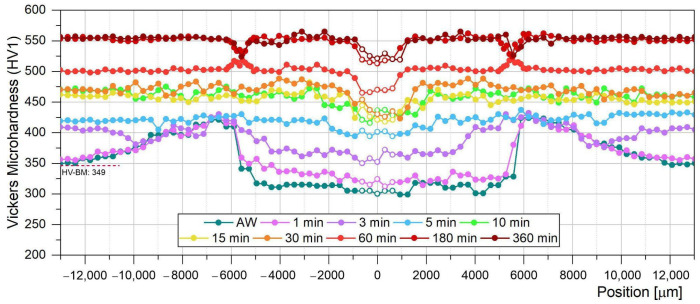
Vickers Microhardness profiles measured in AW and different times of PWHT samples.

Figure 7 shows the microhardness profiles of the welded joint for AW condition as well as for the different PWHT times of permanence at 480 °C (from 1 to 360 min). The blank points correspond to the measurements obtained from the WM (approx. ±2 mm from WCL).

In line with the microstructural changes identified in the AW condition, the microhardness of the BM (349 HV) increases at the SC-HAZ (approx. ±12 mm from WCL) due to the partial ageing of the original martensite. The reached value depends on the position, increasing with the temperature attained during welding up to a maximum of 425 HV at approx. ±6.5 mm from the WCL. In the IC-HAZ (approx. ±6.5 to 4.5 mm from WCL) the local hardness declines up to 315 HV, mainly due to the formation of soft martensite and some γ_r_ also (Figure 4). The R-HAZ (FG-HAZ + CG-HAZ) (approx. ±4.5 to 1 mm from WCL) the microhardness maintains relatively uniform values as a consequence of the fully soft martensite structure, being slightly lower (300 HV) at the WM (±1 mm from WCL).

PWHT produced a hardness increase due to the precipitation of intermetallic phases (Ni_3_Ti, Ni_3_Mo and Fe_2_Mo for longer time periods). It was reported for BM, that precipitation reactions could take place even in periods of less than a minute [11,34].

In the early stages of the PWHT there is a clear difference between the microhardness evolution of each welding zone. In the first 3 min all the zones experienced a hardness increase, except high-temperature SC-HAZ and low-temperature IC-HAZ. After 5 min of permanence the profile was almost uniform, reaching values in the range of 400–430 HV. The WM, CG-HAZ and a local region of SC-HAZ (±9 mm) still showed a lower hardness value (400–410 HV). From 5 min up to 6 h of ageing the hardness increased gradually, achieving a higher homogeneity along the whole welded joint, up to 550 HV approx., except for the WM and the DZ. The CG-HAZ requires a higher PWHT time (60 min) to reach the hardness of the rest of the joint.

DZ presented a local increase of the microhardness values (515–520 HV) for 60 min of PWHT. For 180 and 360 min the peak hardness remained almost constant, but became a soft zone, compared to the rest of the HAZ and BM. These observations were not reported previously. They suggest that the precipitation in this local position is completed after 60 min, while requiring higher times for the other zones. The lower hardness reached at DZ after 180 min could be associated with the presence of the loop-shape (γ_r_) (Figure 6b). Svoboda et al. [13] have also found a local decrease of microhardness in the DZ after a PWHT at 480 °C per 3 h. Feitosa et al. [20] have pointed out that the compositional accumulation of austenite-stabilising elements in the grain boundaries, martensite packets and lath boundaries causes their decreasing into the martensite matrix. Therefore, the lower hardness in the DZ could be associated with a lower density of precipitates.

This is valuable information that was not previously found in the bibliography. These mentioned differences between the microhardness evolution of the different zones of the welded joint are associated with variations in the precipitation and austenite reversion kinetics. The grain size, dislocation density, microsegregation, γ_r_ and partial precipitation that characterise each zone of welded joint microstructure would be responsible for the observed differences.

**Figure 8:**
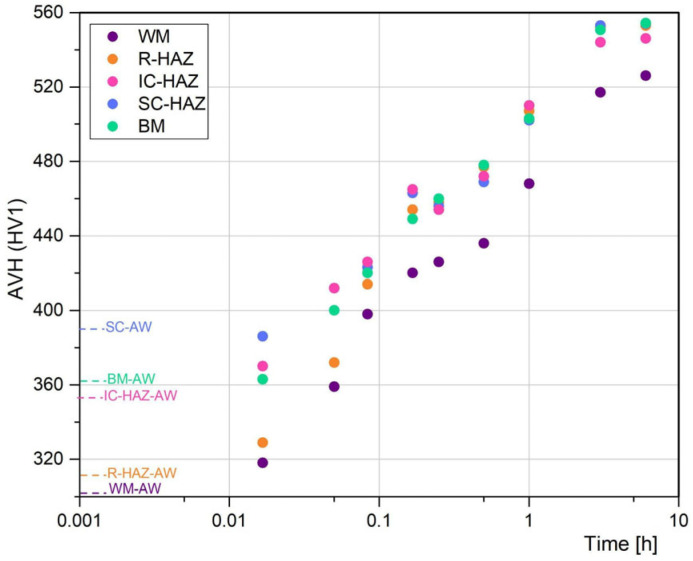
AVH of different weld zones as a function of the PWHT time.

For a better visualisation of the hardness evolution of each zone of the welded joint during the PWHT, the local Average Vickers microhardness (AVH) was determined for each zone: WM, re-crystallized zone (R-HAZ: CG-HAZ + FG-HAZ), IC-HAZ, SC-HAZ and BM. In Figure 8 the AVH of each zone is plotted for different PWHT times.

In AW condition, the WM and R-HAZ had similar AVH due to both experiment complete austenitization and subsequent transformation of soft martensite [11]. BM presented a slightly higher hardness than WM and R-HAZ, which could be associated with the rolling process or some pre-existing precipitation [6,35,36,37]. SC-HAZ zone presented the higher AVH due to the local precipitation achieved during the welding thermal cycle. In the IC-HAZ the steepest hardness gradient was observed, due to the transition between the highest value of the joint at the SC-HAZ (420 HV) to the a low hardness value at R-HAZ (315 HV) in a relatively short distance (1.5 mm).

For 1 min, the R-HAZ and WM show a hardness increase. For 3 min, the evolution of these zones continues, as well as the BM and low-temperature SC-HAZ. High-temperature SC-HAZ remains without hardness variation. At 5 min of permanence BM, SC-HAZ, IC-HAZ and R-HAZ achieve almost the same hardness value, while the WM presented a lower value. For longer PWHT times, this tendency is maintained up to 360 min, measuring a variation of 20–40 HV between the WM and the rest of the weld zones.

Peters and Cupp [34] reported for the BM that merely half a minute of exposure at temperature of 426 and 537 °C produced a hardness increase from 300 HV to 354 and 402 HV, respectively, associated with the minimal incubation time from precipitation as a consequence of the Co content. This is in accordance with the Moshka et al. [5] who observed using TEM hardening precipitates at less than one minute of permanence at ageing temperature, also for BM.

The lower values for the WM could be associated with the presence of the softer γ_r_ islands and possible depleted aged martensite (Figure 5a and Figure 6a) as a consequence of the heterogeneous distribution of the precipitate-forming elements due to microsegregation [11,12]. Also, during welding it is usual to have a loss of metallic elements due to some oxidation [7,38]. Ti is one of the more reactive elements [39]. This could affect the chemical composition of the WM, producing a lower precipitation hardening.

### 3.3. DSC Analysis

**Figure 9:**
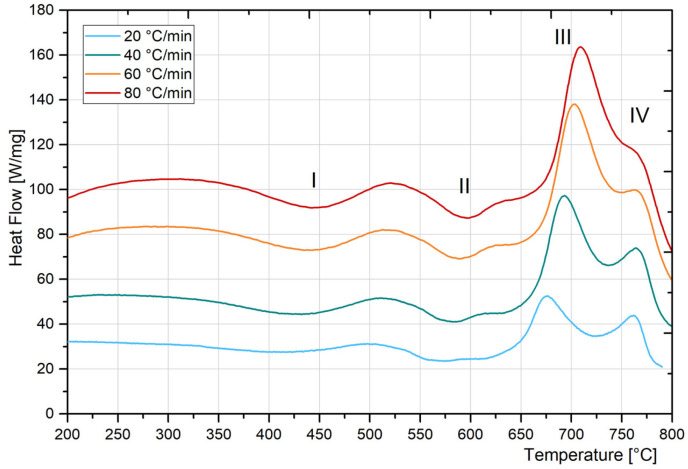
DSC thermograms of MA-C250 steel (BM) at different heating rates.

To analyse the phase transformation in the studied material DSC test was performed in a sample of BM. Figure 9 shows the DSC thermograms obtained for the different analysed heating rates.

There were identified four peaks, related with phase transformations which take place during continuous heating of the material. Table 3 shows the peak and range temperature for each detected reaction, for each heating rate, as well as their respective interpretation according to reported information.

As it was expected, all transformation temperatures showed a slight increase with increasing heating rate [1,9]. There are different interpretations of phase transformations observed at DSC thermograms obtained from a continuous heating of a MA-C250 steel sample in annealed condition. The first exothermic peak (Zone I) is associated with the martensite recovery, the precipitation of carbides or the precipitation of coherent precipitates zones [34,42]. Peters and Cupp [34] have pointed out that this process could only contribute marginally to hardening. The second exothermic reaction (Zone II) has been identified as the main precipitation process (i.e., the formation of intermetallic precipitates) [34,42,43,44,45]. Finally, the endothermic Zone III and Zone IV correspond to the martensite to austenite transformation, that splitted into two steps. The first peak corresponds to the austenite reversion reaction that takes place through a diffusion-controlled process (Zone III) [1,9,31]. Fabian [24] has pointed out, by DSC curve analysis, that the austenite reversion temperature is very close to the end temperature of precipitation and γ_r_ may form during the aged heat treatment. Shamantha et al. [8] have mentioned that when the temperatures are near to A_c1_, the tendency of reversion of martensite is high and the γ_r_ formation could occur. The second step (Zone IV) corresponds to the peak temperature associated with the martensite to austenite transformation by a shear mechanism [9]. This reaction was not sensitive to heating rate, maintaining almost constant the peak temperature. In this sense, the overlap of both reactions increased with heating rate. The analysis of these zones can be useful to understand the phase transformations that take place during the weld thermal cycle, particularly, in the HAZ of the AW condition (Figure 6). The present work could be also useful to improve the interpretation of the phase reactions that take place in MA-C250 steel welds.

### 3.4. Precipitation Reaction Kinetics

The DSC obtained results related with the precipitation peak (Zone II) in a continuous heating condition, at different heating rates, were also used to study the precipitation reaction kinetics in an isothermal condition, following the methodology described before.

**Figure 10:**
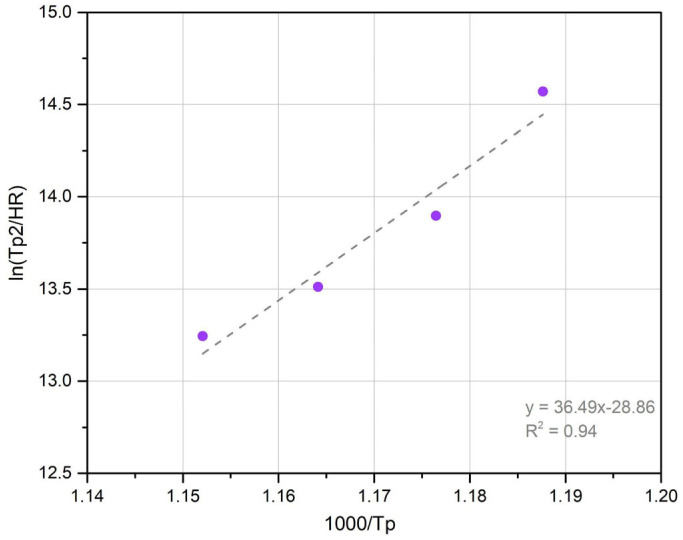
Kissinger plot to determine the activation energy (*E_a_*) for precipitation.

Considering the HR and the Tp obtained for each DSC curve (Zone II), the *E_a_* could be determined from the linear regression (modified Kissinger method) shown in Figure 10.

The *E_a_* for precipitation was 302 kJ/mol, which is similar to reported values (205 kJ/mol) [19], and also considering the lattice diffusion activation energy of Ni (246 kJ/mol), Mo (238 kJ/mol) and Ti (272 kJ/mol) in α-iron [19,47]. Some authors have shown that the *E_a_* for precipitation is usually lower than the one corresponding to the austenite transformation [19,24], which was reported as 342 kJ/mol. Gomes de Carvalho et al. [32] have pointed out that the precipitation and reversion reaction are sensitive to changes in Ni and Co content, even in maraging steels of the same grade. In this sense, the MA-C250 steel reported by Guo et al. [19] has a higher Co content close to 1%, which could explain the difference between the *E_a_* obtained in this work and the reported one.

**Figure 11:**
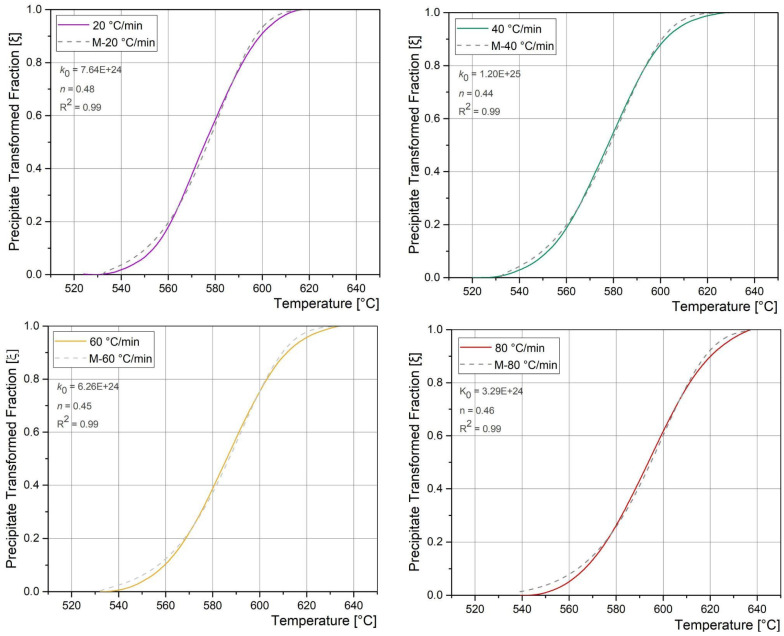
Precipitate transformed fraction vs. Temperature obtained from the DSC results and M-JMAK fitting for the different HR.

The transformed fraction as a function of temperature was obtained from DSC experiments for each HR, considering Equation (1). With the *E_a_* and considering the Equation (3), the experimental results were fitted, obtaining the *k_0_* and *n* values for each HR. Figure 11 shows both the experimental precipitate transformed fraction vs temperature and the fitting performed using the M-JMAK model, for each HR.

A good agreement of the experimental precipitate transformed fraction with the M-JMAK model can be observed. There is an increase of the transformation temperature with increasing heating rates due to the dependence exclusively on diffusion mechanisms [9,31]. The precipitate type could be assumed to be Ni_3_(Mo, Ti) and Fe_2_Mo in only one stage [1,6,8,21]. The *k*_0_ and *n* values, and these are in accordance with the reported range of diffusion controlled growth processes [48,49].

**Figure 12:**
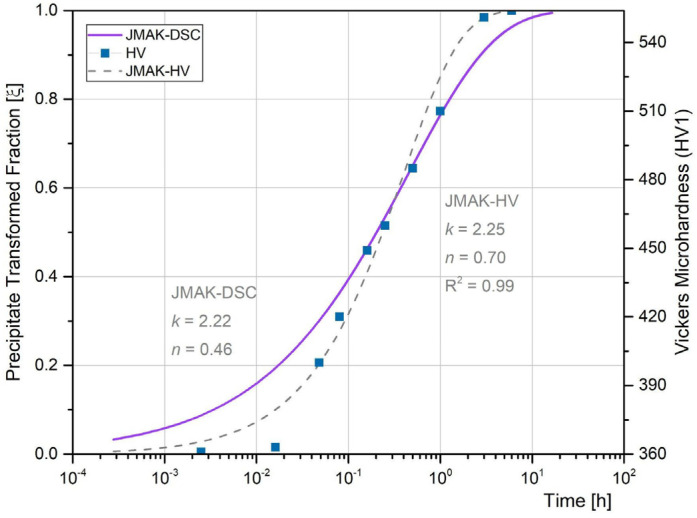
Correlation between the AVH evolution with PWHT time and the obtained JMAK model for precipitation both for the BM at 480 °C.

For each one, the average values (*k*_0_ = 7.3031 × 10^24^ and *n* = 0.46) were used, with the *E_a_*, to determine the reaction rate of the kinetic model for precipitation during isothermal ageing process, following Equation (4). The obtained value for 480 °C was *k* = 2.22. With this and the *n* value, the JMAK model (Equation (4)) was defined. Figure 12 shows the resulting curve, as well as the JMAK fitting (Equation (4)) obtained from the microhardness measurements evolution for BM with PWHT time at 480 °C (AVH-BM).

To apply the JMAK fitting, the AVH corresponding to the AW condition was associated with 0 transformed fraction and the AVH after 6 h of PWHT was considered as fraction 1. The intermediate AVH values for PWHT times were normalized with the minimus and maximus criterion. A linear relationship between AVH and transformed phase fraction was assumed.

As it can be seen, similar results were obtained in both JMAK models, validating the use of Vickers microhardness measurements as an alternative tool to estimate the degree precipitation during PWHT. Several authors have reported *n* values for precipitation reaction of 0.34 [50], 0.35 [34] and 0.39 [41] for 480 °C in a MA-C250 (BM). Guo et al. [19] has been reported for a MA-C250 steel a *n* = 1.46, as a diffusion-controlled growth, mentioning that this value must be improved to reach a better description of the precipitation process in this material. Their results showed that the transformation was complete after an hour, while the peak hardening was reached after 3 hs at the same temperature. On the other hand, Vasudevan et al. [41] have pointed out that the precipitation is completed after 3 h at 482 °C as a consequence of the precipitation of compounds rich in Ti in the early stages and rich in molybdenum towards longer times. This result is consistent with the one obtained in the present work. The same authors have mentioned that the low *n* value could be interpreted in terms of initial precipitation on dislocations followed by growth by a pipe diffusion mechanism. After 1 h, the precipitation reaction tends to slow down due to the nuclei being formed and then they grow, achieving the full transformation after 3 h of exposure to 480 °C [1,34]. The volume fraction of precipitates formed for this material in the mentioned condition could reach 2–3% approx. [19,51].

In relation to the JMAK-HV Mittra et al. [52] in accordance with other authors [48,53] have pointed out that the mechanical properties obtained from hardness measurements might indicate changes in the properties during the different stages of ageing, particularly, when the only phase transformation at the heat treatment temperature is the precipitation [42,54]. In this sense, due to the low fraction of γ_r_ measured in the WM, as a simplification, it was considered that the presence of this phase did not significantly affect the microhardness value of the WM, considering that the microhardness evolution could be associated with the precipitation reaction.

Considering the good agreement found between the JMAK-DSC and JMAK-HV, the different welding zones were analysed using the microhardness evolution to describe the precipitation kinetics during PWHT. In this sense, JMAK models were used to fit the AVH measured values for each zone. Table 4 shows the JMAK parameters obtained from the AVH (Figure 8) fitting, as well as the R^2^ obtained for each zone.

In all cases it can be seen a good agreement of the model used to the experimental data (R^2^ > 0.98). The JMAK results for R and IC-HAZ were similar to the BM one. The reaction rate *k* for SC-HAZ was smaller, which could be related to the pre-existence of precipitates formed during the welding thermal cycle. For WM, the *n* exponent was lower, requiring longer times to complete the reaction. This could be related with the γ_r_ reaction that affects the local chemical composition. Also, as it was mentioned, the chemical composition in the WM could be affected by the oxidising of some elements (Ti, Mo, Ni) in the electric arc. It must be noted that in WM and IC-HAZ there is also a formation or evolution of γ_r_. Nevertheless, due to the very low volume fraction, in this first approximation, its effect on the microhardness evolution was neglected.

### 3.5. Austenite Reversion in the Weld Metal

**Figure 13:**
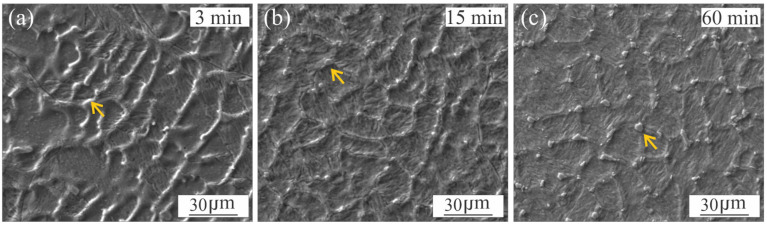
SE-SEM micrograph of γ_r_ in the WM for different PWHT times: 3 min (**a**); 15 min (**b**); 60 min (**c**).

As it was mentioned, as a consequence of the microsegregation occurring at WM during solidification, local variations in Ni, Mo and Ti (Figure 3) induced the γ_r_ formation during PWHT. Floreen et al. [6] have mentioned that Ni tends to stabilize the austenite and Ti tends to destabilise it due to promoting the formation of Ni_3_Ti precipitate type. Mo also contributes indirectly to the γ_r_ nucleation [5]. The kinetics of this transformation and its interaction with precipitation is not clear. Figure 13 shows images of γ_r_ in the WM for different PWHT times.

It can be seen the solidification substructure and the γ_r_ formed at the cell boundary, associated with the microsegregation. It is already present in the microstructure from, at least, 3 min of PWHT, increasing its content with the PWHT time.

**Figure 14:**
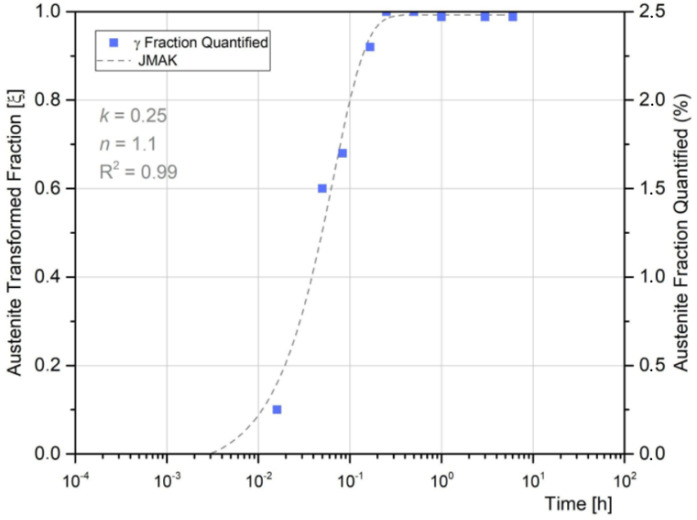
γ_r_ fraction measured in the WM as a function of the PWHT time.

Figure 14 shows the γ_r_ fraction measured in the WM as a function of the PWHT time and the fitting obtained by applying the JMAK model.

A good agreement was obtained between the JMAK model and the experimental data (R^2^ = 0.99). At 15 min, the amount of γ_r_ almost achieved the maximum content, reaching 2.5%, remaining constant up to 360 min. Guo et al. have obtained an *n* value for austenite reversion in C250 maraging steel (BM) of 0.97 [19]. Considering the kinetic theory of Christian [48], the *n* = 1 corresponds to grain boundary nucleation with site saturation. However, although a model has been obtained to study the evolution of reverted austenite during the PWHT in welded joints, due to the limitations of quantitative metallography techniques, more accurate methods should be explored to reach a better quantification of reverted austenite.

Considering both WM transformations (precipitation and reverted austenite) it was observed that γ_r_ starts at very initial stages of PWHT up to 15 min, which means that precipitation occurs mainly after γ_r_ formation has finished. Therefore, the γ_r_ could be responsible for the delay observed in precipitation reaction at WM, compared with BM, due to a decrease in precipitate-forming elements at the martensite matrix [10,55,56].

## 4. Conclusions

In the present work the phase transformation kinetics of GTAW-Pulsed weldments of MA-C250 during PWHT were studied and discussed, improving the comprehension of the microstructural evolution of the different weld zones with PWHT.

After welding the maraging steel in as received condition, in one single pass without filler material, there was obtained an inhomogeneous microhardness profile along the welded joint in AW condition. In the SC-HAZ it was observed a transition from BM (349 HV) to a partial precipitation zone which reached a maximum of 420 HV at the SC/IC-HAZ transition, due to the ageing temperatures attained during welding. In the IC-HAZ the steepest microhardness gradient was measured, where partial aged martensite, soft fresh martensite and some of γ_r_ are present in the microstructure. Within the IC-HAZ there is a narrow DZ in which is observed a loop-shaped orientated phase, associated with γ_r_, which extends to both sides of the DZ. The WM and CG/FG-HAZ showed a lower microhardness (300–310 HV) due to the solubilization produced associated with the high temperatures reached. In the WM, microsegregation of Ti, Mo and Ni was also measured related to the cellular-dendritic solidification mode observed.

During PWHT at 480 °C, the microhardness profile became mostly homogeneous after 5 min of PWHT associated with precipitation reaction. At that time, all the non-melted areas tend to a single average hardness value (430 HV) and evolve jointly until to achieve the maximum average hardness (550 HV). For the WM, after 5 min, the average hardness (390 HV) is less than of the rest of the weldments due to the γ_r_ island formation and Ni, Ti and Mo depleted martensite matrix. The AVH difference between WM and the rest of the weld zones remains constant up to 6 hs. The DZ showed a higher hardness than the rest of the zones for 60 min, reaching its maximum value (520 HV), becoming a soft zone for 3 and 6 hs. In this zone, it was observed a size increase of γ_r_, which could deplete the Ni, Mo and Ti content in the martensite matrix, reducing the precipitation density in this zone.

The DSC technique was used to analyse the phase transformation reaction in continuous heating, at different heating rates in samples of BM. From DSC data the kinetics of precipitation reaction was modelled obtaining the isothermal ageing kinetics at 480 °C, using a JMAK model. The AVH evolution with PWHT time was also fitted with a JMAK model, showing a good agreement with the JMAK obtained from DSC data, validating the use of AVH evolution for monitoring the precipitation reaction during PWHT. The JMAK-HV approach was used to analyse the evolution of the precipitation in the different welding zones during the PWHT.

The microsegregation in the WM promotes the γ_r_ formation at the higher local composition sites during PWHT, reaching a maximum of 2.5% after 15 min at 480 °C. A JMAK expression was obtained to model the γ_r_ evolution during PWHT. The presence of γ_r_ at the very early stages could explain the delay observed in the precipitation reaction in WM.

## Figures and Tables

**Table 1:** Chemical composition of MA-C250 steel.

(wt%)	C	Mn	Si	Ni	Mo	Co	Ti	Al	P	S	Fe
MA-C250	0.01	0.04	0.09	18.2	4.9	7.9	0.4	0.15	0.009	0.003	Bal
ASTM-A538	0.03 *	0.10 *	0.10 *	17–19	4.6–5.2	7–8.5	0.3–0.5	0.05–0.15	0.01 *	0.01 *	Bal

* maximum value.

**Table 2:** GTAW-P welding parameter.

I_p_ [A]	I_b_ [A]	I_RMS_ [A]	V_RMS_ [V]	d_e-p_ [mm]	F [Hz]	Ws [mm/s]	HI [J/mm]
120	72	99	10	1	15	4	246

I_p_: Peak current; I_b_: Base current; I_RMS_: Root Mean Square Current; V_RMS_: Root Mean Square Voltage; d_e-p_: electrode-piece distance; Ws: Welding speed.

**Table 3:** Analysis of transformations detected in DSC runs.

Vc [°C/min]	Zone *	Peak Temp. [°C]	Temp. Range [°C]	Possible Transformation
20	I Exo	429	212–513	Recovery of martensite and formation of carbides (minor hardening) [33] Formation of coherent precipitation zones [40,41,42]
40		437	255–517	
60		446	293–524	
80		451	315–531	
20	II Exo	571	516–614	Formation of the main strengthening precipitates [42,43,44,45,46,47]
40		577	521–628	
60		586	532–631	
80		595	539–637	
20	III Endo	686	624–730	Reverted Austenite formation [34,40,42,43,44,45]
40		693	633–737	
60		702	647–749	
80		711	659–756	
20	IV Endo	771	732–797	Martensite to austenite by shear [42,46]
40		769	741–823	
60		772	751–825	
80		768	751–821	

* Endo: Endothermic; Exo: Exothermic.

**Table 4:** JMAK parameters obtained from AVH fitting for the different weld zones.

Zone	*n*	*k*	R^2^
WM	0.57	2.4	0.99
R-HAZ	0.75	2.2	0.99
IC-HAZ	0.72	3.2	0.98
SC-HAZ	0.78	1.7	0.99

## Data Availability

Data is contained within the article.

## Abbreviations

GTAW-P	Gas Tungsten Arc Welding—Pulsed
AW	As Welded
WM	Weld Metal
R-HAZ	Recrystallized Heat Affected Zone
CG-HAZ	Coarse Grain Heat Affected Zone
FG-HAZ	Fine Grain Heat Affected Zone
IC-HAZ	Intercritical Heat Affected Zone
SC-HAZ	Subcritical Heat Affected Zone
DZ	Dark Zone
BM	Base Material
PWHT	Post Weld Heat Treatment
WCL	Weld Central Line
DSC	Differential Scanning Calorimetry
EDS	Energy Dispersive Spectroscopy
JMAK	Johnson-Mehl-Avrami-Kolmogorov
M-JMAK	Modified Johnson-Mehl-Avrami-Kolmogorov
γ_r_	Reverted Austenite
MA	Maraging
SRM	Solid Rocket Motor
AR	As Received
HV	Hardness Vickers
AVH	Average Vickers Hardness
HI	Heat Input
HT	Heat Treatment
*E_a_*	Activation Energy
*T_p_*	Temperature of the precipitation peak
*HR*	Heating Rate
*n*	Avrami Exponent
*k*_0_	Reaction Rate
*k*	Constant
*T*_0_	Onset Temperature

## References

[1] Sha W., Malinov S., Sha W., Guo Z. (2009). Maraging Steels–Modelling of Microstructure, Properties and Applications.

[2] Sundaresan S., Manirajan M., Rao B.N. (2010). On the Fracture Toughness Evolution in Weldments of Maraging Steel Rocket Motor Case. Mater. Des..

[3] Freeman R., Chaturvedi M. (2020). New Welding Techniques for Aerospace Materials. Welding and Joining for Aerospace Materials.

[4] Kumar B.D., Nayana B.S., Shree D.S. (2016). Design and Structural Analysis of Solid Rocket Motor Casing Hardware Used in Aerospace Applications. J. Aeronaut. Aerosp. Eng..

[5] Moshka O., Pinkas M., Brosh E., Ezersky V., Meshi L. (2015). Addressing the Issue of Precipitates in Maraging Steels—Unambiguous Answer. Mater. Sci. Eng. A.

[6] Floreen S. (1968). The Physical Metallurgy of Maraging Steel. Metall. Rev..

[7] Lang F.H., Kenyon N., Lang F.H., Kenyon N. (1971). Welding of Maraging Steel.

[8] Shamantha C., Narayanan R., Iyer K., Radhakrishnan V., Seshadri S., Sundararajan S., Sundaresan S. (2000). Microstructural Changes During Welding and Subsequent Heat Treatment of 18Ni (250-grade) Maraging Steel. Mater. Sci. Eng. A.

[9] Kapoor R., Kumar L., Batra I. (2003). A Dilatometric Study of the Continuous Heating Transformations in 18 wt.% Ni Maraging Steel of Grade 350. Mater. Sci. Eng. A.

[10] Li K., Shan J., Wang C., Tian Z. (2016). Influence of Aging Temperature on Strength and Toughness of Laser-Welded T-250 Maraging Steel Joint. Mater. Sci. Eng. A.

[11] Gupta R.N., Raja V. (2020). Environmentally Assisted Cracking Susceptibility of Dark Etched HAZ/Parent Metal Interface Region of 18Ni 250 Maraging Steel Weldment. Mater. Sci. Eng. A.

[12] Rao V.V., Reddy G.M., Raju A.V.S. (2010). Influence of Post-weld Heat Treatments on Microstructure and Mechanical Properties of Gas Tungsten Arc Maraging Steel Weldments. Mater. Sci. Technol..

[13] Svoboda H.G., Duran M., Belzunce F.J., Rodríguez C. (2024). Estimation of Local Mechanical Properties by Small Punch Test in Welded Joints of Maraging C250 Steel. J. Mater. Eng. Perform..

[14] Ahmed B., Tariq F., Naz N., Baloch R.A. (2012). How Multiple Weld Repairs Impact Maraging Steel. Weld. J..

[15] Shamantha C., Narayanan R., Iyer K., Radhakrishnan V., Seshadri S., Sundararajan S., Sundaresan S. (2000). Tensile Properties and Fracture Toughness of 18Ni (250 grade) Maraging Steel Weldments. Sci. Technol. Weld. Join..

[16] Jose B., Manoharan M., Natarajan A., Muktinutalapati N.R., Reddy G.M. (2022). Development of a Low Heat-Input Welding Technique for Joining Thick Plates of 250 Grade Maraging Steel to Fabricate Rocket Motor Casing. Mater. Lett..

[17] Sakai P., Lima M., Fanton L., Gomes C., Lombardo S., Silva D., Abdalla A. (2016). Comparison of Mechanical and Microstructural Characteristics in Maraging 300 Steel Welded by three different processes: LASER, PLASMA and TIG. Procedia Eng..

[18] Tariq F., Baloch R.A., Ahmed B., Naz N. (2009). Investigation into Microstructure of Maraging Steel 250 Weldments and Effect of Post-Weld Heat Treatments. J. Mater. Perform..

[19] Guo Z., Sha W., Li D. (2004). Quantification of Phase Transformation Kinetics of 18 wt. % Ni C250 Maraging Steel. Mater. Sci. Eng. A.

[20] Feitosa A.L.M., Escobar J., Ribamar G.G., Avila J.A., Padilha A.F. (2022). Direct Observation of Austenite Reversion During Aging of 18Ni (350 Grade) Maraging Steel Through In-Situ Synchrotron X-Ray Diffraction. Met. Mater Trans A.

[21] Conde F., Escobar J., Oliveira J., Jardini A., Filho W.B., Avila J. (2019). Austenite Reversion Kinetics and Stability During Tempering of an Additively Manufactured Maraging 300 Steel. Addit. Manuf..

[22] Gao P., Jing G., Lan X., Li S., Zhou Y., Wang Y., Yang H., Wei K., Wang Z. (2021). Effect of Heat Treatment on Microstructure and Mechanical Properties of Fe-Cr-Ni-Co-Mo Maraging Stainless Steel Produced by Selective Laser Melting. Mater. Sci. Eng. A.

[23] dos Santos L.P.M., Béreš M., de Castro M.O., Sarvezuk P.W.C., Wu L., Herculano L.F.G., Paesano A., Silva C.C., Masoumi M., de Abreu H.F.G. (2020). Kinetics of Reverted Austenite in 18 wt.% Ni Grade 300 Maraging Steel: An in-Situ Synchrotron X-Ray Diffraction and Texture Study. Miner. Met. Mater. Soc..

[24] Fabian R.J. (2022). Phase Transformation Kinetics in Laser-Powder Bed Fused Fe-Cr.Ni-Al-Maraging Stainless Steel. Master’s Thesis.

[25] Bai Y., Wang D., Yang Y., Wang H. (2019). Effect of Heat Treatment on the Microstructure and Mechanical Properties of Maraging Steel by Selective Laser Melting. Mater. Sci. Eng. A.

[26] Lupi G., Bettini E., Deirmina F., Casati R. (2024). Microstructural and Mechanical Properties of a Novel Cobalt and Titanium Free Maraging Steel for Laser Powder Bed Fusion. J. Mater. Res. Technol..

[27] Murthy C., Krishna A.G., Reddy G. (2019). Microstructural and Mechanical Properties and Dissimilar Metal Gas tungsten Constricted Arc Welds: Maraging Steel to 13-8 Mo Stainless Steel. Def. Technol..

[28] American Society for Testing and Materials (2021). E415-21 Standard Test Method for Analysis of Carbon and Low Alloy Steel by Spark Atomic Emission Spectrometry.

[29] Teixeira F.R., Scotti F.M., Jorge V.L., Scotti A. (2023). Combined Effect of the Interlayer with Travel Speed on Features of Thin Wall WAAM Under Two Cooling Approaches. Int. J. Adv. Manuf. Technol..

[30] Sastry K.Y., Narayanan R., Shamantha C.R., Sundaresan S., Seshadri S.K., Radhakrishnan V.M., Iyer K.J.L., Sundararajan S. (2003). Stress Corrosion Cracking of Maraging Steel Weldments. Mater. Sci. Technol..

[31] Rohit B., Muktinutalapati N.R. (2017). Austenite Reversion in 18% Ni Maraging Steel and its Weldments. Mater. Sci. Technol..

[32] de Carvalho L.G., Andrade M.S., Plaut R.L., Souza F.M., Padilha A.F. (2013). A Dilatometric Study of the Phase Transformations in 300 and 350 Maraging Steels During Continuous Heating Rates. Mater. Res..

[33] da Fonseca D.P.M., Feitosa A.L.M., de Carvalho L.G., Plaut R.L., Padilha A.F. (2020). A Short Review on Ultra-High-Strength Maraging Steels and Future Perspectives. Mater. Res..

[34] Peters D.T., Cupp C.R. (1965). The Kinetics of Aging Reaction in 18 Pct Ni Maraging Steels. Transit. Metall. Soc. AIME.

[35] Jacob K., Yadav D., Dixit S., Hohenwarter A., Jaya B.N. (2021). High Pressure Torsion Processing of Maraging Steel 250: Microstructure and Mechanical Behaviour Evolution. Mater. Sci. Eng. A.

[36] Freitas G.H.d.O., de Oliveira C.A.S. (2018). Effect of Hot Deformation on Microstructure, Hardness and Precipitation Kinetics in a 350 Maraging Steel Modified by Titanium Alloy. Mater. Res..

[37] Floreen S., Decker R.F., Decker R.F. (1979). Source Book on Maraging Steel.

[38] Gupta R.N., Raja V.S., Mukherjee M.K., Murty S.V.S.N. (2017). On Improving the Quality of Gas Tungsten Arc Welded 18Ni 250 Maraging Steel Rocket Motor Casing. Miner. Met. Mater. Soc. ASM Int..

[39] Evans G.M., Bailey N., Greening G. (1997). Metallurgy of Basic Weld Metal.

[40] Viswanathan U., Banerjee S., Krishnan R. (1988). Effects of Aging on the Microstructure of 17-4 PH Stainless Steel. Mater. Sci. Eng. A.

[41] Vasudevan V.K., Kim S.J., Wayman C.M. (1990). Precipitation Reactions and Strengthening Behavior in 18 Wt Pct nickel maraging steels. Met. Trans. A.

[42] Bui N., Dabosi F. (1972). Contribution to the Study of the Effect of Molybdenum on the Ageing Kinetics of Maraging Steels. Cobalt.

[43] Habiby F., ul Haq A., Hashmi F.H., Khan A.Q. (1986). Lattice changes in the martensitic phase due to ageing in 18 wt% nickel maraging steel grade 350. Proceedings of the International Conference on Martensitic Transformations (ICOMAT-86).

[44] Goldberg A., O‘Connor D.G. (1967). Influence of Heating Rate on Transformations in an 18 per cent Nickel Maraging Steel. Nature.

[45] Goldberg A., Decker R.F. (1979). Maraging Steels.

[46] Saul G., Roberson J.A., Adair A.M., Decker R.F. (1979). Maraging Steels.

[47] Brandes E.A., Brook G.B., Heinemann B., Brook G.B. (1999). Smithells Metals Reference Book.

[48] Christian J.W. (2002). Chapter 12: Formal Theory of Transformation Kinetics. Part 1: The Theory of Transformation in Metals and Alloys.

[49] Krishtal M.K. (1970). Diffusion Processes in Iron Alloys.

[50] Mahadevan S., Jayakumar T., Rao B., Kumar A., Rajkumar K., Raj B. (2008). X-Ray Diffraction Profile Analysis for Characterizing Isothermal Aging Behavior of M250 Grade Maraging Steel. Miner. Met. Mater. Soc. ASM Int..

[51] Zhu F., Yin Y.F., Faulkner R.G. (2011). Microstructural Control of Maraging Steel C300. Mater. Sci. Technol..

[52] Mittra J., Kulkarni U., Dey G. (2009). Hardness Based Model for Determining the Kinetics of Precipitation. Mater. Sci. Eng. A.

[53] Guo Z., Sha W. (2002). Quantification of Precipitation Hardening and Evolution of Precipitates. Mater. Trans..

[54] Sinha I., Mandal R. (2011). Avrami Exponent Under Transient and Heterogeneous Nucleation Transformation Conditions. J. Non-Cryst. Solids.

[55] Reddy G.M., Rao V.V., Raju A.V.S. (2009). The Effect of Post-weld Heat Treatments on the Microstructure and Mechanical Properties of Maraging Steel Laser Weldments. J. Mater. Des. Appl..

[56] Abreu H.F., Silva J.J., Silva M.R., da Silva M.J.G. (2015). Influence of Reverted Austenite on the Texture and Magnetic Properties of 350 Maraging Steel. J. Magn. Magn. Mater..

